# Blinded gait assessment in idiopathic normal pressure hydrocephalus: reliability and correlation with clinical and patient-reported outcomes

**DOI:** 10.1186/s12987-025-00704-2

**Published:** 2025-09-08

**Authors:** Maria Ekblom, Dag Nyholm, Lena Zetterberg, Katarina Laurell, Johan Virhammar

**Affiliations:** 1 Department of Medical Sciences, Neurology, Uppsala University, Uppsala, Sweden; 2 Department of Women’s and Children’s Health, Physiotherapy, Uppsala University, Uppsala, Sweden; 3 Department of Biomedical and Clinical Sciences, Neurology, Linköping University, Linköping, Sweden

## Abstract

**Background:**

Idiopathic normal pressure hydrocephalus (iNPH) predominantly manifests with gait disturbances, yet clinical assessments are vulnerable to confirmation bias, particularly post-shunt surgery. Blinded video evaluations are a method to enhance objectivity in gait assessment, but their reliability has never been systematically investigated. The aim was to evaluate the inter-rater reliability of blinded gait assessments in iNPH patients and to investigate how these assessments correlate with the Hellström iNPH scale and patient-reported health status following shunt surgery.

**Methods:**

Thirty-nine patients (mean age 75.5 years) diagnosed with iNPH between 2019 and 2023 were recorded performing Timed Up and Go (TUG) test before and after shunt surgery. Patients who required a walking aid were excluded. Four specialized raters, blinded to timepoint, evaluated gait pattern and graded improvement. Inter-rater agreement was quantified by Krippendorff’s α; Spearman’s ρ assessed correlations between graded improvement, Hellström iNPH scale changes, and EuroQol 5-Dimension 5-Level Visual Analogue Scale (EQ-VAS) differences.

**Results:**

Agreement on video graded improvements was strong (α = 0.80, 95% CI: 0.76–0.84), whereas agreement on specific gait patterns was moderate (α = 0.53, 95% CI: 0.43–0.62). Graded improvement scores correlated moderately with changes in the Hellström iNPH scale (ρ = 0.67, *p* < 0.01) and showed fair correlation with EQ-VAS (ρ = 0.37, *p* < 0.01).

**Conclusions:**

Blinded video assessments reliably captured postoperative gait improvements in iNPH and showed strong inter-rater agreement. While specific gait pattern ratings were less consistent, combining structured video scoring with clinical scales can improve outcome evaluation. More refined tools are needed to better detect subtle changes in gait and to reflect patient-perceived recovery.

**Supplementary Information:**

The online version contains supplementary material available at 10.1186/s12987-025-00704-2.

## Introduction

Idiopathic normal pressure hydrocephalus (iNPH) is a neurological disorder that affects elderly individuals. Its exact cause remains unclear, but it is characterized by enlarged ventricles in the brain without an increase in intracranial pressure. The standard treatment for iNPH is shunt surgery to drain the excess cerebrospinal fluid [1, 2].

iNPH leads to a triad of symptoms: impairment in gait and cognition, and urinary incontinence, with gait disturbance being the most prominent and distinctive symptom of the disease [2, 3]. Gait abnormalities in iNPH are often described as a broad-based stance, shuffling steps, and pronounced difficulty in executing turns [4]. The presence of two or more gait irregularities typical for iNPH suggests a probable diagnosis according to international guidelines [2].

The gradual onset and slow progression of symptoms often mask the early recognition of iNPH [5]. Gait disturbances often serve as the earliest clinical indicator, and the ability to walk unaided is essential for autonomy, directly influences the patient’s overall quality of life (QoL) [6, 7]. Therefore, evaluation of gait disturbances is critical both in the diagnostic work-up and for assessing postoperative outcomes.

Current gait assessment methods often involve visually evaluating the patient’s gait as they walk a predetermined distance. These assessments can be influenced by prior knowledge, such as diagnostic imaging results (e.g., MRI showing enlarged ventricles), which may lead to confirmation bias. This bias is particularly problematic in postoperative evaluations, where healthcare providers may unconsciously expect improvements due to the surgical intervention, potentially overestimating its effectiveness.

Although the absence of blinding can compromise objectivity, the impact of blinded assessments of gait in iNPH has not been systematically evaluated. This study aimed to evaluate the inter-rater reliability of blinded gait assessments in detecting changes in gait patterns among patients with iNPH. Additionally, the study sought to examine whether observable changes in gait correlate with other clinical measurements and patient-reported outcomes following shunt surgery.

## Method

### Study population

This study included patients diagnosed with iNPH between 2019 and 2023 at a single center. All patients were diagnosed according to international guidelines [2] and assessed by a specialized iNPH team, including neurologists, neurosurgeons, physiotherapists, occupational therapists, and radiologists. All patients had undergone cerebrospinal fluid (CSF) tap test and shunt surgery following clinical evaluation. Additionally, video recordings of their gait were conducted as part of routine clinical practice, both prior to surgery and during follow-up visits.

Patients were considered for shunt surgery at our center based on an overall clinical assessment, including the presence of typical symptoms, radiological findings, CSF tap test, and lumbar infusion test. The tap test and infusion test were used as supplementary predictive tools; negative results did not preclude patients from undergoing shunt surgery.

Patients were excluded if video recordings were incomplete (e.g., missing turning or sit-to-stand segments) or if they used any walking aid either before or after shunt surgery.

### Measurements & procedures

To assess the change in clinical symptoms of individuals with iNPH, the Hellström iNPH scale [8] was utilized. This scale evaluates the domains corresponding to the clinical manifestations of iNPH: gait, balance, cognition, and urinary incontinence. Besides quantitative measurements, it also includes a qualitative assessment of the gait pattern (Table 1). The scores from these domains are combined into a composite score ranging from 0 to 100, where a score of 0 indicates significant impairment due to symptoms, and 100 represents the functional level of healthy age-matched controls.

Patients were evaluated using the Hellström iNPH scale at two time points: before CSF tap test (baseline) and at 3 months after shunt surgery (follow-up). Changes in scores between baseline and follow-up were compared to assess clinical improvement.

Patients were video recorded while performing the Timed Up and Go (TUG) test, a standardized clinical assessment of gait and mobility in older adults [9], at baseline and follow-up.

To assess the QoL of the patients, the EuroQol 5-Dimension 5-Level Visual Analogue Scale (EQ-VAS) [10] was utilized. This scale allows patients to rate their overall health on a scale from 0 to 100, where 0 represents the worst imaginable health and 100 indicates the best possible health state. Patients were asked to complete the EQ-VAS at baseline and follow-up.

### Blinded gait assessment

Four raters were selected to perform the blinded gait assessment. Two raters (II and IV) were neurologists subspecialized in hydrocephalus, while the remaining two (I and III) included a neurologist and a physiotherapist, both specialized in movement disorders. The raters were blinded to whether the film was recorded at baseline or at follow-up, with the recordings randomized and labeled “A” and “B” to conceal the sequence.

To further ensure blinding, the videos were processed to obscure contextual information that could reveal the setting in which the recordings were made. This included blurring the background to prevent recognition of whether the recording was pre- or post-surgery and obscuring any individuals walking next to the patient to minimize risk of falling.

The raters underwent a standardized training protocol prior to the assessments to ensure consistent use of the grading scales. As part of the training, they reviewed video recordings from three separate patients, each shown with paired films labeled “1” and “2”. These pairs represented distinct levels of postoperative change: one with clear improvement, one with moderate observable improvement, and one with small observable improvement. This process was intended to familiarize raters with how different degrees of gait change would manifest on video and to calibrate their scoring accordingly.

The raters used a structured rating scale assessment when grading the videos (Supplementary Data 1). Each rater began by viewing Film A and grading the patient’s gait pattern using an ordinal gait scale (Table 1) from the Hellström iNPH-scale [8]. Next, they viewed Film B, applying the same gait scale for evaluation. Additionally, they evaluated the change in gait pattern between Film A and Film B (unaware of which of the videos that was recorded pre- and postoperative) using the video outcome scale, an 11-point Likert scale ranging from − 5 (“much worse”) to + 5 (“much better”) which was developed for this study (Table 2).

**Table 1:** The ordinal gait scale used to assess the gait pattern

Grading
1 Normal
2 Slight disturbance of tandem walk and turning
3 Wide-based gait with sway, without foot corrections
4 Tendency to fall, with foot corrections
5 Walking with cane
6 Bimanual support needed
7 Aided
8 Wheelchair bound

**Table 2:** The video outcome scale used to assess the graded improvement

Gradings
+ 5 Much better
+ 4
+ 3
+ 2
+ 1
0 Unchanged
-1
-2
-3
-4
-5 Much worse

### Data analysis

All statistical analyses were performed using IBM SPSS Statistics (Version 28.0) [11]. Inter-rater agreement for gait pattern (ordinal gait scale) and graded improvement (video outcome scale) was assessed using Krippendorff’s alpha (α) [12, 13].

To evaluate change over time, the difference in scores on the Hellström iNPH scale [8] and the EQ-VAS [10] was calculated and Wilcoxon’s signed rank test was used to assess the magnitude of change between baseline and follow-up. Spearman’s rank correlation coefficients (ρ) [14, 15] were used to examine relationships between the change in graded improvement (difference between Film A and B) and changes in the Hellström iNPH scale and EQ-VAS scores. Correlations were analyzed separately for each rater, as well as the mean of the correlation coefficients across all raters to assess overall trends.

### Ethics

This study was approved by the Regional Ethical Review Board, Swedish Ethical Review Authority (dnr: 2023-03078-02). No additional data were collected beyond routine clinical practice. All data were pseudonymized prior to analysis to ensure patient confidentiality.

## Results

A total of 39 patients (mean age: 75.5 years; range: 57–88) were included. All had been diagnosed with probable or possible iNPH and had undergone shunt surgery (Table 3). There were no dropouts during the study period; all included patients completed both baseline and follow-up assessments. Wilcoxon signed rank test revealed significant changes to both the iNPH score (Z = -4.11, *p* < 0.001) and EQ-VAS (Z = -2.91, *p* = 0.004) from baseline to follow-up at group level, (Table 4).

**Table 3:** Patient demographic data

	Values
Number of participants	39
Age, mean (range)	75.5 years (57–88)
Sex distribution (%)	25 males (64%), 14 females (36%)
iNPH-score (IQR) baseline	55.6 (49.1–64.1)
iNPH-score (IQR) follow-up	63.3 (54.3–75.4)
EQ-VAS (IQR) baseline	50.0 (45.0–65.0)
EQ-VAS (IQR) follow-up	67.5 (50.0-76.3)
Time from tap test to surgery, months (IQR)	10.0 (6.0-18.5)
Time between film A (tap test) and film B (follow-up), months (IQR)	13.0 (10.3–21.8)

Abbreviations: EQ-VAS: EuroQol visual analogue scale range; iNPH-score: Total score on the Hellström iNPH scale

### Agreement between raters

The agreement between raters for gait pattern, assessed via the ordinal gait scale (Table 1), was moderate, with α = 0.53 (95% CI: 0.43–0.62). In contrast, agreement between raters for graded improvement, assessed via the video outcome scale (Table 2), was strong, α = 0.80 (95% CI: 0.76–0.84) (Table 4).

**Table 4:** Krippendorff’s alpha for blinded assessment of gait pattern and graded improvement

Assessment Type	Krippendorff’s Alpha (α)	Confidence Intervals (95%)
Gait Pattern	0.53	0.43–0.62
Graded Improvement	0.80	0.76–0.84

### Correlation between outcome scales

**Fig. 1:**
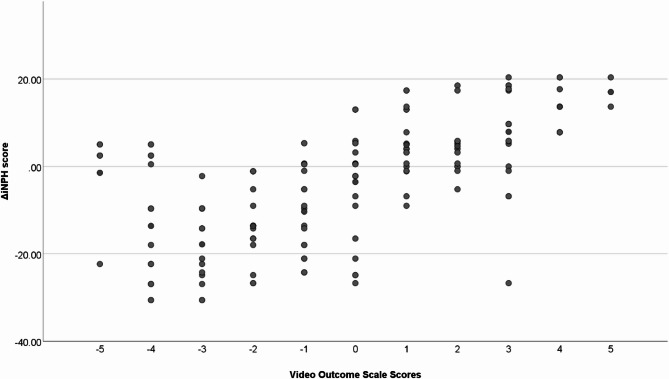
Scatterplot between raters’ scores on the video outcome scale and the Hellström iNPH scale [8]

Spearman’s rank correlation analysis revealed significant relationships between scores of graded improvements, using the video outcome scale, and the Hellström iNPH scale [8] (mean ρ = 0.67, *p* < 0.01), with correlation coefficients for each rater ranging from 0.64 to 0.71 (*p* < 0.001) (Fig. 1).

Fair to moderate correlations were observed between the individual scores of graded improvement in gait and change in EQ-VAS. Significant correlations were identified for Raters I and II (ρ = 0.48 and ρ = 0.42, *p* < 0.05), whereas graded improvement scores for Raters III and IV did not correlate significantly with EQ-VAS (ρ = 0.32 and ρ = 0.33, *p* > 0.05). The mean correlation across all raters was fair (ρ = 0.37, *p* < 0.01).

A moderate correlation was identified between change in EQ-VAS and the Hellström iNPH scale scores (ρ = 0.51, *p* < 0.01).

## Discussion

This study demonstrates that blinded gait assessments can reliably estimate postoperative improvements in gait among patients with iNPH.

The inter-rater agreement for graded improvement scores was strong, indicating high consistency among raters in assessing overall improvement and suggests that clinicians, even from different professional backgrounds, were able to consistently evaluate postoperative changes in gait when blinded to timepoint. This supports the feasibility of using structured video assessments as a robust outcome tool in both clinical and research settings. The strong agreement also highlights the utility of the video outcome scale (Table 2), which may have provided sufficient sensitivity to detect subtle gait improvements not easily captured by broader categorical tools.

There was a greater variability observed among raters when assessing specific gait characteristics. This variability may be attributed to factors such as subjective interpretation of gait patterns and the inherent complexity of assessing individual gait components.

Rating gait patterns using video analysis was employed by Ishikawa et al. [16] who analyzed inter-rater agreement on various gait characteristics in patients with iNPH. They found excellent agreement on two out of six gait characteristics (α = 0.80). The current study focused on analyzing the overall gait pattern rather than individual gait characteristics, which may have contributed to the lower agreement observed. This could be due to the fact that not all gait characteristics are present in every patient or that raters may prioritize different aspects of gait when forming their assessments, leading to variability in their ratings.

The lower agreement in the current study may also be explained by differences in how the ordinal gait scale (Table 1), used to assess gait pattern, was applied. The ordinal gait scale consists of eight levels, four of which evaluate the qualitative gait features observed when patients walk unaided. While the scale incorporates the classic gait disturbances of iNPH, it may not fully account for the less pronounced characteristics [17–19]. Notably, categories 3 and 4 (wide-based gait vs. tendency to fall) may be difficult to distinguish, especially if a patient presents with both a broad-based gait and subtle balance instability.

To evaluate consistency in rating gait patterns among specialists, a subset analysis using Cohen’s kappa was conducted on the two raters specialized in iNPH (rater II and IV), yielding a moderate agreement (κ = 0.44, *p* < 0.001). This agreement was notably lower than that reported by Virhammar et al. [20] where inter-rater reliability between two observers was very good (κ = 0.74).

In the study by Virhammar et al. [20], the entire range of the ordinal gait scale was utilized, and patients using walking aids were also included, making it easier to differentiate between categories. In contrast, this study only applied four levels of the ordinal gait scale, focusing on patients who walked unaided. This limited scoring range may have contributed to the lower inter-rater variability observed.

In clinical practice, diagnosing subtle gait changes is challenging, particularly due to the variability in gait presentation among patients [17]. To address these limitations, alternative or weighted scoring systems could be applied to existing gait scales to improve sensitivity and reliability, especially in patients with milder impairments. These findings also highlight an important issue: that the gait pattern in iNPH can be inherently difficult to categorize using the ordinal gait scale, particularly when patients walk unaided.

In this study, patients reliant on walking aids were excluded to avoid potential distortion of gait patterns caused by assistive devices such as walkers or canes. While walking aids are beneficial for compensating mobility impairments, they have been shown to alter natural gait mechanics, potentially obscuring underlying gait characteristics [21–23]. This exclusion criterion allowed for a more accurate evaluation of typical iNPH-related gait patterns, thereby improving the internal validity of the findings. However, this also resulted in a study population skewed toward milder or moderately affected individuals (ordinal gait scale levels 1–4), limiting the generalizability of the results to patients with more severe gait dysfunction.

While our study set out to investigate the usefulness of grading gait pattern and postoperative improvement, the findings indicate that detailed assessments of gait did not achieve higher inter-rater reliability than the global impression-based graded improvement scale. General assessments of overall improvement may offer a more robust and clinically practical alternative, particularly in settings where simplicity and reproducibility are prioritized.

Spearman rank correlation analysis revealed a moderate-to-strong correlation between the Hellström iNPH scale and the graded improvement scores for respective rater, while slightly lower correlations were observed between the EQ-VAS and the iNPH scale. This suggests that subjective perceptions of recovery, as measured by the EQ-VAS, may capture broader aspects of health and QoL beyond those assessed by clinical rating scales.

Previous studies have shown that symptom improvement following shunt surgery is associated with enhanced QoL [24–26]. However, outcomes may be influenced by the timing of surgical intervention. Patients with iNPH who experience longer waiting times before surgery tend to have poorer clinical outcomes compared to those who are treated earlier [27, 28]. A study by Chidiac et al. [29] recommended that surgery should be performed within 3 months of decision for a more favorable outcome. In our study, the median waiting time was 10 months.

This extended delay may have influenced patients’ subjective perceptions of recovery due to lingering symptoms, such as limitations of daily activities, which are not fully reflected in the iNPH scale. This could explain the weaker correlation observed between the EQ-VAS and graded improvement, as well as the moderate correlation between the iNPH scale and EQ-VAS.

Patients in this study also reported relatively low scores at EQ-VAS at baseline, with a median score of 50. This is slightly lower than in previous studies [25, 28] but consistent with the findings of Israelsson et al. [7]. Lower QoL in iNPH patients is well-documented, particularly when compared to population-based reference groups [24, 28, 30]. QoL can also be influenced by factors such as comorbidities, social support, depressive symptoms, and limitations in daily activities [28, 30–32], which are not fully captured by scales currently employed in clinical practice.

### Limitations

The sample size in this study was relatively small, which may limit the generalizability of the findings. While the study provides valuable insights into the inter-rater reliability of blinded gait assessments in iNPH patients, a larger cohort would be valuable to confirm these results.

The study utilized video recordings to analyze gait, a practical approach that is easily implemented in a clinical setting. However, while video analysis is useful for observing gait parameters, more advanced and robust methods could be employed to improve the precision and depth of gait analysis, such as accelerometer [33], motion capture systems [34], or deep learning algorithms [35]. The downside to these advanced methods, however, is their limited availability and practicality for routine clinical use. In contrast, analyzing video recordings requires only a brief rater training session, and the scoring process itself is straightforward and time-efficient. This makes the method feasible in selected settings that support standardized follow-up and have the capacity for basic video documentation.

As with many elderly patients, patients with iNPH may suffer from other comorbidities in addition to the iNPH diagnosis. While comorbidities were not systematically assessed in this study, many iNPH patients are likely to present with age-related conditions such as polyneuropathy, vascular changes, or musculoskeletal issues (e.g., joint stiffness or arthritis). These conditions can independently affect gait performance and balance, potentially confounding the observed improvements attributed to shunt surgery. Furthermore, such comorbidities may influence subjective assessments of recovery, including EQ-VAS scores, by shaping patients’ perceptions of their overall health status. The absence of standardized comorbidity data is therefore a limitation that could have impacted both the objective gait analysis and the patient-reported outcomes.

Additionally, the study’s reliance on two time points (before and after surgery) may not capture the full trajectory of patient recovery or the long-term effects of shunt surgery. A longer follow-up period with multiple assessment points might provide a more comprehensive understanding of patient outcomes.

## Conclusions

This study demonstrated strong inter-rater agreement in assessing graded improvements, which moderately correlated with established clinical outcome scales. While moderate agreement was observed in gait pattern evaluations, this may reflect the inherent subjectivity of such assessments, particularly among patients with milder impairments. These findings suggest that general assessments of gait improvement may currently be more reliable and clinically practical than detailed gait pattern scoring. Nevertheless, structured rating scales can still provide valuable complementary information, particularly when used alongside established outcome measures such as the Hellström iNPH scale. Together, these tools may enhance the evaluation of treatment response following shunt surgery and support more standardized follow-up. Future research should explore factors affecting quality of life to better understand postoperative recovery in iNPH patients.

## Supplementary Information

Below is the link to the electronic supplementary material.

Supplementary Material 1

## Data Availability

No datasets were generated or analysed during the current study.
