# Supplementary material for: Blinded gait assessment in idiopathic normal pressure hydrocephalus: reliability and correlation with clinical and patient-reported outcomes
Source: Fluids Barriers CNS. 2025 Sep 8;22:91. doi: 10.1186/s12987-025-00704-2 (PMC12418656; doi:10.1186/s12987-025-00704-2)
Supplement: Supplementary file 1 — Supplementary Material 1 [file 12987_2025_704_MOESM1_ESM.docx]

**Gait Assessment and Video Outcome Scale**

| Gait assessment according to the iNPH scale | |
| --- | --- |
| 🞎 | Normal. |
| 🞎 | Slight disturbance of tandem walking and/or turning. |
| 🞎 | Wide-based gait and with sway, without foot corrections. |
| 🞎 | Tendency to fall, with foot correction. |

| Assessment of walking ability in Film B,  compared to Film A | | |
| --- | --- | --- |
| 🞎 | +5 | Much better |
| 🞎 | +4 |  |
| 🞎 | +3 |  |
| 🞎 | +2 |  |
| 🞎 | +1 |  |
| 🞎 | 0 | Unchanged |
| 🞎 | -1 |  |
| 🞎 | -2 |  |
| 🞎 | -3 |  |
| 🞎 | -4 |  |
| 🞎 | -5 | Much worse |
